# Safety and efficacy of vernakalant for the conversion of atrial fibrillation to sinus rhythm; a phase 3b randomized controlled trial

**DOI:** 10.1186/s12872-016-0289-0

**Published:** 2016-05-28

**Authors:** Gregory N. Beatch, Brian Mangal

**Affiliations:** Cardiome Pharma Corp., 1441 Creekside Drive 6th Floor, Vancouver, BC V6J 4S7 Canada; PAREXEL International Corp., Lowell, MA USA

**Keywords:** Atrial fibrillation, Antiarrhythmic, Cardioversion, Vernakalant

## Abstract

**Background:**

Atrial fibrillation (AF) is a common cardiac arrhythmia that is associated with significant health risks. One strategy to mitigate the risks associated with long-term AF is to convert AF to sinus rhythm (SR). This study assessed the efficacy and safety of vernakalant hydrochloride for the pharmacological conversion of AF to SR.

**Methods:**

Patients with recent-onset (duration >3 h– ≤ 7 days) symptomatic AF and no evidence or history of congestive heart failure were randomized in a 2:1 ratio to receive vernakalant or placebo. Patients received an infusion of vernakalant (3 mg/kg) or placebo over 10 min, followed by a second infusion of vernakalant (2 mg/kg) or placebo 15 min later if AF had not been terminated. The primary efficacy endpoint was conversion of AF to SR for at least 1 min within 90 min of the start of drug infusion. The primary safety endpoint was a composite of: occurrence of clinically significant hypotension, clinically significant ventricular arrhythmia (including torsades de pointes, ventricular tachycardia or ventricular fibrillation) or death within 2 h of starting the drug infusion.

**Results:**

A total of 217 patients were randomized to receive vernakalant (*n* = 145) or placebo (*n* = 72). Of the 129 individuals who received vernakalant, 59 (45.7 %) converted to SR compared with one of the 68 patients (1.5 %) who received placebo (*p* < 0.0001). Conversion to SR was significantly faster with vernakalant than with placebo (*p* < 0.0001), and a greater proportion of patients who received vernakalant than those who received placebo reported no AF-related symptoms at 90 min (*p* = 0.0264). The primary composite safety endpoint was observed in one patient receiving vernakalant and in no patients receiving placebo. In the vernakalant arm, dysgeusia, paraesthesia and sneezing were the most common treatment-emergent adverse events, and three serious adverse events occurred that were considered to be related to study drug.

**Conclusions:**

Vernakalant resulted in rapid cardioversion of recent-onset AF in almost half of the study population and was generally well tolerated. The safety outcomes affirmed the need for careful selection and management of haemodynamically stable candidates for cardioversion.

**Trial registration:**

NCT00989001.

**Electronic supplementary material:**

The online version of this article (doi:10.1186/s12872-016-0289-0) contains supplementary material, which is available to authorized users.

## Background

Atrial fibrillation (AF) is the most common arrhythmia encountered in clinical practice; projections suggest 3.3 million adults in the USA will have AF by 2020 [1]. Although not directly life-threatening, AF may be a marker of mortality and increases cardiovascular morbidity; AF is associated with an increased long-term risk of stroke and heart failure [2]. A desired strategy to mitigate some of the risks associated with AF remains the conversion of AF to normal sinus rhythm (SR). This conversion can be achieved via electrical cardioversion (ECV) or pharmacological cardioversion, both of which are widely used [3]. While ECV is generally more efficacious than pharmacological cardioversion, it can be associated with dermal injury, arrhythmias, thromboembolic complications and early recurrence of AF, and patients must be anaesthetized during the procedure [4–8]. At the time of the present clinical trial, the available pharmacological agents had been shown to have limited efficacy in converting AF to SR and to have the potential for causing serious arrhythmias or hypotension [5, 9, 10].

Vernakalant hydrochloride is a novel, and relatively atrial-selective, antiarrhythmic agent that rapidly and durably converts recent-onset AF to SR [11, 12]; it has been shown to be more effective than amiodarone for rapid conversion of AF of 3–48 h’ duration [13]. Previous randomized, double-blind, placebo-controlled trials have demonstrated that, compared with placebo, intravenous vernakalant effectively converts AF to SR in patients with recent-onset AF (duration >3 h– ≤ 7 days; *p* < 0.001 in both Arrhythmia Conversion Trial [ACT] I and ACT III) [12, 14]. These studies also identified that patients with low baseline systolic blood pressure (SBP) and congestive heart failure (CHF) receiving vernakalant may be at an increased risk of hypotension [15]. This randomized, double-blind, placebo-controlled phase 3b clinical trial investigated the safety and efficacy of vernakalant hydrochloride for the conversion of symptomatic recent-onset AF to SR in patients with no history or evidence of CHF.

## Methods

### Study design

This multicentre study involved 113 sites in Canada, Chile, Israel, Mexico, Peru, South Africa and the USA; 58 of the 113 sites enrolled at least one patient. The study was conducted under the terms of a Special Protocol Assessment (SPA) procedure (Guidance for Industry Special Protocol Assessment, May 2002); comments received from the US Food and Drug Administration (FDA) and subsequent revisions to the study protocol were addressed and clearly documented as part of the formal SPA process. The protocol and amendments to the protocol were reviewed and approved by an Independent Ethics Committee or Institutional Review Board at each study site. The study was overseen by the sponsors, an independent Data Safety Monitoring Board (DSMB) and Clinical Events Committee (CEC), and was conducted in accordance with the Declaration of Helsinki, Good Clinical Practice and International Council for Harmonisation guidelines, as well as applicable laws and regulations. All included patients provided written informed consent.

### Patient screening and selection

Patients who were candidates for participation in the study were assessed before enrolment, within 12 h before dosing. Screening included assessment of inclusion and exclusion criteria. Included patients were adults aged 18 − 85 years with recent-onset (duration >3 h– ≤ 7 days) symptomatic AF for whom best management was determined by the investigator to be acute cardioversion to SR. Patients were also required to be adequately hydrated (as determined by the investigator). If AF had continued for more than 48 h, patients were to be managed in accordance with the standard of care for anticoagulation, as recommended by the American College of Cardiology/American Heart Association/European Society of Cardiology guidelines [16]. Patients were excluded if they had evidence or a history of heart failure or evidence of left ventricular dysfunction, heart rate less than 50 beats per minute (bpm) or symptomatic bradycardia. Additional inclusion and exclusion criteria were the same as those detailed in Roy et al. [12]. For example, patients were excluded if they met any of the following criteria: had a QRS interval of more than 0.14 s without a pacemaker; an uncorrected QT interval of more than 0.44 s; acute coronary syndrome or myocardial infarction; or cardiac surgery performed in the 30 days before planned enrolment [12].

### Treatment plan

Patients were randomized in a 2:1 ratio to receive vernakalant or placebo. Participants received either a 10-min intravenous infusion of vernakalant (3 mg/kg) or an equivalent volume of placebo (normal saline). They were then observed for 15 min; if patients were in AF or atrial flutter at the end of the observation period, a second 10-min infusion of vernakalant (2 mg/kg) or equivalent volume of placebo was administered, unless any dose-stopping criteria had been met. Dose-stopping criteria were similar to those listed in Roy et al. [12] and comprised: an uncorrected QT interval of more than 0.55 s or QT interval prolonged by more than 25 % compared with the baseline value; a heart rate of 40–50 bpm alongside symptoms of bradycardia, or a heart rate less than 40 bpm; SBP less than 85 mmHg; new bundle-branch block; QRS interval prolonged by more than 50 % compared with baseline; asymptomatic ventricular tachycardia lasting 30 s or longer; symptomatic ventricular tachycardia; ventricular fibrillation; torsades de pointes of any duration; sinus pause of 5 s or longer; complete heart block; or intolerable side effects as assessed by the investigator.

ECV was permitted 2 h after the start of the first infusion of study drug if the patient was still in AF or atrial flutter. Patients were observed in hospital for a minimum of 4 h after the start of infusion of the study drug. Vital signs were recorded at screening, baseline, every 5 min from the start of infusion until 1 h post-infusion, then every 30 min until 4 h post-infusion, and at 24 h and 1 week post-infusion. Continuous Holter monitoring was performed from 30 min before the start of the infusion until 24 ± 4 h later. Several 12-lead electrocardiograms (ECGs) were recorded, including at screening and baseline, at the start and end of the infusion(s), at AF conversion, every 30 min from 1 to 4 h post-infusion, and at 24 h and 1 week post-infusion. In patients in whom ECV was attempted, additional monitoring of vital signs and ECGs was carried out between the time that conscious sedation was initiated and 2 h after the last electrical shock.

### Study outcomes

The primary efficacy endpoint was successful conversion to SR for at least 1 min within 90 min of the start of the first infusion. The main outcome variable was the proportion of patients achieving this endpoint based on independent CEC assessment of ECG/Holter data. Secondary efficacy variables were the time between first exposure to study drug and conversion of AF to SR, and the proportion of patients reporting no AF symptoms at 90 min after first drug exposure. Exploratory efficacy variables included the proportion of patients who converted to SR within 90 min and maintained SR for 24 h following first exposure to the study drug (sustained conversion rate), and the impact of symptoms of AF on quality of life at 90 min after first drug exposure.

The primary safety outcome was the proportion of patients who met any of the following three criteria, as assessed by the independent CEC: clinically significant hypotension; clinically significant ventricular arrhythmia; or death within 2 h of the start of exposure to study drug. Clinically significant hypotension was defined as any of the following: SBP of less than 90 mmHg with treatment with pressors, SBP of less than 90 mmHg with albumin, dextran or hydroxyethyl starch treatment, or SBP of less than 90 mmHg and seizures. Ventricular arrhythmia was defined as any of the following: sustained ventricular tachycardia (lasting ≥30 s on ECG, telemetry or Holter monitoring) with a heart rate of at least 120 bpm, torsades de pointes with a duration of 10 s or more on Holter or 12-lead ECG, or ventricular fibrillation of any duration.

### Statistical analysis

All randomized patients who received any amount of study drug were included in the efficacy and safety analyses. All analyses were performed using Statistical Analysis Systems 9.2 (SAS® Institute, Cary, NC, USA). Baseline demographics and patient characteristics were summarized by treatment group. For continuous data, summaries included the number of observations, and mean, standard deviation (SD) and median values. For categorical data, frequency counts and percentages were presented.

A sample size of 450 patients was planned, based on the primary safety endpoint. The primary safety objective of the study was to demonstrate a less than 1 % risk of clinically significant events with vernakalant, based on requirements set by the US FDA. With 300 patients treated with vernakalant and no events, the risk of clinically significant events would be less than 1 %, as defined by the one-sided upper 95 % confidence interval (C.I) for the vernakalant arm. To prevent any bias, a placebo group of 150 participants was planned. This total sample size of 450 patients would have provided more than 98 % power to detect a treatment difference of 35 % in the primary efficacy endpoint, assuming a cardioversion rate of less than 10 % in the placebo group. Estimation of the C.I for the primary safety endpoint was based on the exact approach.

The primary efficacy endpoint was analysed using the Cochran–Mantel–Haenszel test stratified by geographic region. If the primary efficacy endpoint demonstrated a statistically significant positive finding, each of the secondary efficacy endpoints was analysed. For the time to conversion of AF to SR, the Kaplan–Meier method was used to obtain the distribution of the time to conversion associated with each treatment group. The two treatment groups were then compared using a two-sided log-rank test. For the proportion of patients reporting no symptoms at 90 min, data were analysed using a two-sided *χ*^2^ test. Analyses of the exploratory efficacy variables were conducted using two-sided *χ*^2^ tests.

## Results

Between 28 October 2009 and 21 October 2010, 217 patients were enrolled in the study and randomized to receive either vernakalant (*n* = 145) or placebo (*n* = 72). The majority of randomized patients (197 of 217) received at least one infusion of study drug and were included in the efficacy and safety analyses. Patient enrolment was suspended and the study was placed on clinical hold on 21 October 2010, after a single patient in the vernakalant arm experienced the primary safety outcome. Following a meeting with the US FDA in December 2011, it was determined by the sponsors that this study would not meet regulatory expectations. Therefore, the study was terminated early.

### Baseline demographics and characteristics

Demographics, medical history and characteristics of AF were similar in the two treatment groups (Table 1). Over half of the patients were from Israel (37.1 %) or the USA (24.4 %), and the majority were white (95.4 %) and male (61.4 %). Patients had a mean (± SD) age of 62.7 (±13.2) years, and a mean weight of 87.6 (±17.7) kg. Cytochrome P450 2D6 (CYP2D6) genotype status was known in 51.8 % of patients, most of whom were classified as extensive (57.8 %) or intermediate (31.4 %) metabolizers. AF and medical history were similar in patients in the two treatment groups. Most patients (60.9 %) had experienced at least one previous AF episode, and 16.8 % had experienced more than three previous episodes. The median duration of patients’ current AF episode was 25 h.

**Table 1:** Baseline patient demographics and characteristics

Demographics and characteristics	Placebo (*n* = 68)	Vernakalant (*n* = 129)
Sex, n (%)		
Male	45 (66.2)	76 (58.9)
Female	23 (33.8)	53 (41.1)
Race, n (%)		
White	65 (95.6)	123 (95.3)
Black or African–American	0	5 (3.9)
Asian	2 (2.9)	0
American Indian or Alaskan Native	0	1 (0.8)
Other	1 (1.5)	0
Country, n (%)		
Israel	24 (35.3)	49 (38.0)
USA	17 (25.0)	31 (24.0)
South Africa	15 (22.1)	21 (16.3)
Canada	9 (13.2)	20 (15.5)
Chile	2 (2.9)	3 (2.3)
Mexico	1 (1.5)	4 (3.1)
Peru	0	1 (0.8)
Age, years, mean (SD)	60.8 (14.1)	63.7 (12.7)
Weight, kg, mean (SD)	89.8 (17.6)	86.5 (17.7)
Classification based on CYP2D6 genotype, n (%)		
Extensive metabolizer	17 (25.0)	42 (32.6)
Intermediate metabolizer	13 (19.1)	19 (14.7)
Poor metabolizer	0	4 (3.1)
Ultra-rapid metabolizer	0	2 (1.6)
Indeterminate	1 (1.5)	4 (3.1)
Missing	37 (54.4)	58 (45.0)
Number of previous AF episodes, n (%)		
0	24 (35.3)	53 (41.1)
1	14 (20.6)	32 (24.8)
2	15 (22.1)	15 (11.6)
3	2 (2.9)	9 (7.0)
>3	13 (19.1)	20 (15.5)
Duration of current AF episode, hours, mean (SD)	41.0 (36.3)	37.3 (36.6)
Duration of current AF episode, n (%)		
1 day	28 (41.2)	62 (48.1)
2 days	16 (23.5)	25 (19.4)
3 days	8 (11.8)	13 (10.1)
4 days	5 (7.4)	8 (6.2)
5 days	1 (1.5)	7 (5.4)
6 days	6 (8.8)	3 (2.3)
7 days	0	2 (1.6)
Missing data	4 (5.9)	9 (7.0)
Medical history, n (%)		
Hypertension	39 (57.4)	89 (69.0)
Hyperlipidaemia	39 (57.4)	62 (48.1)
Valvular heart disease	13 (9.1)	27 (20.9)
Diabetes mellitus	16 (23.5)	18 (14.0)
Ischaemic heart disease	12 (17.6)	18 (14.0)
Percutaneous transluminal coronary angioplasty	9 (13.2)	18 (14.0)
Syncope, fainting or postural hypotension	10 (14.7)	9 (7.0)
Myocardial infarction	7 (10.3)	11 (8.5)
Angina pectoris	3 (4.4)	10 (7.8)
Atrial flutter	3 (4.4)	8 (6.2)
Coronary artery bypass graft	3 (4.4)	5 (3.9)
Pacemaker	3 (4.4)	3 (3.2)
Ventricular tachycardia	2 (2.9)	1 (0.8)
Symptoms of AF, n (%)		
Palpitations	*n* = 66	*n* = 128
	42 (63.6)	88 (68.8)
Fatigue	*n* = 65	*n* = 128
	33 (50.8)	61 (47.7)
Chest pain	*n* = 66	*n* = 128
	5 (7.6)	17 (13.3)
Dyspnoea	*n* = 66	*n* = 128
	10 (15.2)	26 (20.3)
Dizziness	*n* = 66	*n* = 128
	13 (19.7)	20 (15.6)

*AF* atrial fibrillation, *SD* standard deviation

### Efficacy

**Fig. 1:**
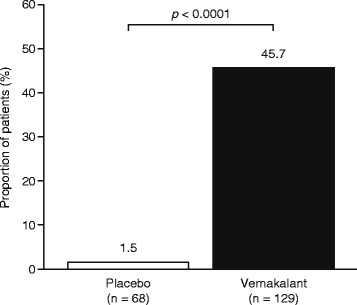
Proportion of patients who converted to sinus rhythm. Proportion of patients who converted to sinus rhythm within 90 min of the start of the first infusion of placebo or vernakalant

**Fig. 2:**
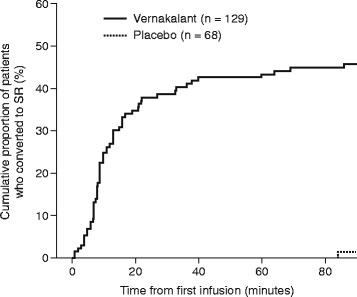
Time to cardioversion. Kaplan–Meier plot of the time to conversion from atrial fibrillation to sinus rhythm within 90 min of the start of the first infusion of placebo or vernakalant

Vernakalant met the primary and secondary efficacy endpoints; a significantly greater proportion of patients in the vernakalant group than in the placebo group converted from AF to SR in the 90 min following first drug exposure (45.7 % [*n* = 59 of 129] vs 1.5 % [*n* = 1 of 68], respectively; *p* < 0.0001; Fig. 1). Conversion to SR was significantly faster in the vernakalant group than in the placebo group (*p* < 0.0001; Fig. 2); 25 % of patients receiving vernakalant had converted to SR by 11 min, and the patient receiving placebo who met the primary efficacy endpoint converted to SR after 84 min.

The proportion of patients reporting no AF symptoms at 90 min was significantly greater in the vernakalant group than in the placebo group (47.3 % vs 30.9 %, respectively; *p* = 0.0264). In both treatment groups, the proportion reporting AF symptoms was reduced at 90 min relative to baseline. The degree to which symptoms of AF impacted on quality of life had improved in patients who received vernakalant compared with those who received placebo (*p* = 0.0227) by 90 min. After 24 h, 56 of the 59 patients (94.9 %) who received vernakalant and who had met the primary efficacy endpoint were still in SR, as was the patient who had converted to SR after receiving placebo.

### Safety

The primary composite safety endpoint was observed in one patient receiving vernakalant who experienced hypotension and ventricular arrhythmia within 2 h of treatment initiation, and in no patients in the placebo group. The primary safety endpoint was also observed within 2 h of ECV in a patient who had previously received vernakalant. The patient developed hypotension 16 h after the last vernakalant infusion: this was not considered by the investigator to be related to study drug.

The incidence of treatment-emergent adverse events (AEs) was higher in the vernakalant group than in the placebo group. Treatment-emergent AEs that occurred within 24 h in more than 3 % of patients are listed in Table 2. The majority of treatment-emergent AEs occurring within 24 h of the start of infusion and considered to be related to study drug were observed in the vernakalant group, with dysgeusia (14.7 %), sneezing (7.8 %) and paraesthesia (6.2 %) being the most common.

**Table 2:** Frequent treatment-emergent adverse events

Adverse event, n (%)	Placebo (*n* = 68)	Vernakalant (*n* = 129)
Dysgeusia	1 (1.5)	19 (14.7)
Sneezing	0	11 (8.5)
Paraesthesia	1 (1.5)	8 (6.2)
Headache	2 (2.9)	7 (5.4)
Hypertension	3 (4.4)	6 (4.7)
Hypotension	3 (4.4)	5 (3.9)
Nausea	1 (1.5)	5 (3.9)
Increased blood pressure	0	4 (3.1)
Ventricular tachycardia	0	4 (3.1)
Bradycardia	0	4 (3.1)
Pain	3 (4.4)	0

Treatment-emergent adverse events that occurred in more than 3 % of patients within 24 h of placebo or vernakalant administration

The incidence of serious AEs (SAEs) occurring within 24 h of the start of study drug infusion was similar in the two treatment groups (Table 3); SAEs occurred in 4 of 68 patients (5.9 %) who received placebo and 10 of 129 patients (7.8 %) who received vernakalant. SAEs that occurred in more than one patient in the 24 h following initiation of infusion included atrial fibrillation (two patients in the placebo group and six patients in the vernakalant group) and atrial flutter (two patients in the vernakalant group). The proportion of patients with SAEs that occurred between 24 h and 30 days after study drug infusion was similar in both treatment groups: 14 of 68 patients (20.6 %) who received placebo and 22 of 129 patients (17.1 %) who received vernakalant. SAEs that occurred in more than one patient in a treatment group were atrial fibrillation (9 placebo, 16 vernakalant), atrial flutter (0 placebo, 2 vernakalant) and non-cardiac chest pain (2 placebo, 1 vernakalant). Three patients had SAEs considered to be related to vernakalant; all occurred within 2 h of dosing and led to discontinuation of treatment (nausea, headache, confusional state and cold sweat in one patient, sinus arrest in another patient and cardiogenic shock in the third patient). The number of discontinuations due to AEs was low and similar in the two groups.

**Table 3:** Serious adverse events

Serious adverse event, n (%)	Placebo (*n* = 68)	Vernakalant (*n* = 129)
Any SAE	4 (5.9)	10 (7.8)
Blood and lymphatic system disorders		
Coagulopathy	0	1 (0.8)
Iron-deficiency anaemia	1 (1.5)	0
Cardiac disorders		
Atrial fibrillation	2 (2.9)	6 (4.7)
Atrial flutter	0	2 (1.6)
Cardiogenic shock	0	1 (0.8)
Sinus arrest	0	1 (0.8)
Gastrointestinal disorders		
Nausea	0	1 (0.8)
General disorders and administration site conditions		
Non-cardiac chest pain	1 (1.5)	0
Hepatic failure	0	1 (0.8)
Infections and infestations		
Sepsis	0	1 (0.8)
Musculoskeletal and connective tissue disorders		
Rhabdomyolysis	0	1 (0.8)
Nervous system disorders		
Headache	0	1 (0.8)
Psychiatric disorders		
Confusional state	0	1 (0.8)
Renal and urinary disorders		
Acute renal failure	0	1 (0.8)
Respiratory, thoracic and mediastinal disorders		
Pneumonia aspiration	0	1 (0.8)
Skin and subcutaneous tissue disorders		
Cold sweat	0	1 (0.8)

Serious adverse events that occurred within 24 h after patients received vernakalant or placebo

There were two deaths in the study: one in the vernakalant group and one in the placebo group. The patient in the vernakalant group who died was a 77-year-old man. He was genotyped as a CYP2D6 extensive metabolizer and had plasma levels of vernakalant within the expected range. He had a medical history of hypertension and chronic alcohol abuse. The patient was receiving concomitant medication, including enalapril and acetylsalicylic acid, and had had dyspnoea for approximately 1 week, persistent palpitations for 48–72 h and AF for approximately 48 h before he presented to the hospital. Assessment of AF symptoms during screening and at baseline revealed the presence of palpitations and fatigue, but no chest pain, dyspnoea or dizziness. The patient’s blood pressure was 139/76 mmHg and oxygen saturation while breathing room air was 96 %. A two-dimensional echocardiogram performed before randomization while the individual was in AF with a ventricular rate of 156 bpm revealed diffuse hypokinesis with an ejection fraction of 44 %, and moderate left ventricular hypertrophy. He experienced cardiogenic shock with symptoms of sweating and nausea, together with decreased blood pressure and heart rate, beginning 10 min after the start of vernakalant infusion. Approximately 4 h after the onset of cardiogenic shock, the patient was electrically cardioverted to SR. From day 2 to day 10, the patient had an SAE of rhabdomyolysis, two SAEs of electromechanical dissociation, and AEs of gastritis and encephalopathy. Additional SAEs reported and resulted in a fatal outcome; these included: coagulopathy, aspiration pneumonia, hepatic failure, acute renal failure and sepsis (all beginning on day 2); anaemia (starting on day 4); gastrointestinal haemorrhage (starting on day 12); ischaemic colitis (starting on day 17); and hypovolemic shock (starting on day 29). All of these subsequent AEs and SAEs were assessed by the investigator as not being related to study drug. The patient died on day 29.

The patient in the placebo group who died was an 84-year-old woman with a history of valvular heart disease, hypertension, hyperlipidaemia, coronary artery bypass graft surgery, myocardial infarction, a brain arteriovenous fistula and a cerebrovascular accident. She was receiving concomitant medication, including clopidogrel, hydrochlorothiazide and olmesartan. The patient received two full infusions of placebo, did not convert to SR and did not undergo ECV. Oral sotalol was administered approximately 3 h after the last infusion of placebo, and the patient spontaneously converted to SR 2 h later. The patient had an SAE of cerebrovascular accident 9 days later and, on the same day, she experienced respiratory failure, cardiovascular arrest and acute renal failure; these SAEs were all assessed by the investigator as not being related to study drug. The patient died due to the cerebrovascular accident, approximately 7 h after this event.

## Discussion

Intravenous vernakalant is approved in European and other countries as a relatively atrial-selective antiarrhythmic drug for the conversion of recent-onset AF to SR. This phase 3b study aimed to evaluate the safety and efficacy of vernakalant compared with placebo in patients with recent-onset (duration >3 h– ≤ 7 days) symptomatic AF and no evidence or history of CHF. Due to the small number of CYP2D6 poor metabolizers enrolled, it was not possible to determine from the data whether CYP2D6 genotype had any influence on the occurrence of adverse events in this study.

The study met all of the primary and secondary efficacy objectives; treatment with vernakalant resulted in a significantly greater proportion of patients converting from AF to SR within the first 90 min (45.7 % vs 1.5 %, respectively) and at a faster conversion rate than with placebo. Most participants (94.9 %) who converted to SR reported sustained conversion at 24 h. Furthermore, a greater proportion of patients in the vernakalant group than in the placebo group reported no AF symptoms at 90 min. The speed and durability of conversion in this study are consistent with the results of all previous studies of intravenous vernakalant for patients with recent-onset AF [12–14, 17–20].

In terms of safety, vernakalant was well-tolerated in most patients. The primary composite safety endpoint was observed in one patient receiving vernakalant and in no patients in the placebo group. One patient also met the criteria for hypotension within 2 h of ECV, 16 h after receiving vernakalant, but this was not considered by the study investigator to be related to study drug. The incidence of treatment-emergent AEs was higher in the vernakalant group than in the placebo group; the most commonly reported treatment-emergent AEs (dysgeusia, sneezing and paraesthesia) were the same as those reported in previous trials [12, 14]. Drug-related SAEs occurred in three patients in the vernakalant group; one of these events, cardiogenic shock, was unexpected and led to the suspension of study enrolment. In addition, the United States Investigational New Drug Application was placed on clinical hold by the US FDA. This patient subsequently experienced a series of AEs and SAEs and died from lower gastrointestinal bleeding and multi-organ failure. Although the independent DSMB recommended that the trial continue after reviewing these data, it was terminated by the sponsors [21].

It may be worth reviewing the safety events in this study in the context of cardioversion of AF in clinical practice. While ECV is generally regarded as an effective treatment with a good safety profile, recently published large registry studies have allowed a more systematic evaluation. Pisters et al. reported on 1801 patients undergoing cardioversion at enrolment into the multicentre prospective Euro Heart Survey on AF [6]. Major complications in the peri-procedural period in the ECV group included: non-sudden cardiac death (2 patients [0.3 %]), sick sinus syndrome (5 patients [0.7 %]), ventricular tachycardia (6 patients [0.8 %]), torsades de pointes (1 patient [0.1 %]), ventricular fibrillation (3 patients [0.4 %]), asystole (2 patients [0.3 %]), cardiac syncope (1 patient [0.1 %]), new heart failure (7 patients [1.1 %]), transient ischaemic attack (2 patients [0.3 %]), stroke (2 patients [0.3 %]) and major bleeding patients (9 [1.3 %]). Grönberg et al. reported on the immediate arrhythmic complications following 6906 ECVs performed in 2868 patients with AF of less than 48 h’ duration in the Finnish CardioVersion (FinCV) study conducted between 2003 and 2010 [22]. In total, 63 ECVs (0.9 %) resulted in bradyarrhythmia, defined as asystole lasting longer than 5 s and/or bradycardia of less than 40 bpm. A further analysis of the retrospective data from the FinCV study was performed by Nuotio et al. [23]. In addition to reporting that increasing age, female sex, diabetes mellitus and heart failure were risk factors for thromboembolism, these authors reported that time to cardioversion was potentially an important new determinant of stroke risk in patients with AF of less than 48 h’ duration who were not being treated with anticoagulants (*N* = 5116). It was reported that a delay in time to cardioversion of 12 h or longer from symptom onset was associated with a 1.1 % risk of thromboembolic complications; when the duration of AF was less than 12 h, the risk of thromboembolism was almost fourfold lower (0.3 %). Further studies are required to confirm these findings, however, and to establish whether the same risks apply to patients undergoing pharmacological cardioversion.

Crijns et al. recently reported on a prospective international, multicentre, observational cardioversion study: the International Registry on Cardioversion of Atrial Fibrillation (RHYTHM-AF) [3]. A total of 3940 consecutive patients with recent-onset AF considered for cardioversion were enrolled between May 2010 and June 2011. Overall, cardioversion was performed at a median (interquartile range) of 4.0 (1.4–25.5) hours after admission (after 6 [2.4–40.0] hours for ECV [*n* = 1946] and after 1.7 [0.6–8.7] hours for pharmacological cardioversion [*n* = 1026]); no cardioversion was performed in an additional 968 patients. One patient in each group died and one individual in each group experienced heart failure within 5 days of admission. In the 5–70-day follow-up period, there were 6 (0.31 %), 7 (0.68 %) and 9 (0.93 %) deaths, as well as 8 (0.41 %), 4 (0.39 %) and 4 (0.41 %) incidents of heart failure, in the ECV, pharmacological cardioversion and no cardioversion groups, respectively.

Taken together, these studies have revealed that there is a background level of serious outcomes, including death, occurring in patients with AF who are candidates for cardioversion, whether cardioversion is attempted by electrical or pharmacological means, and even when cardioversion is not attempted at all. While confirmation in prospective randomized trials is necessary, the study by Nuotio et al. [23] is also suggestive of a potential benefit of rapid cardioversion (within 12 h) of onset of AF in order to reduce the risk of thromboembolism in patients with AF of less than 48 h’ duration. Vernakalant provides a median time to conversion of 11 min and may be valuable for the cardioversion of haemodynamically stable patients with recent-onset AF.

## Conclusions

The data from this phase 3b study confirm that vernakalant rapidly and effectively converts recent-onset AF to SR in patients without CHF, and provides symptom relief. Vernakalant was generally well tolerated, although there was a higher incidence of AEs in the vernakalant group than in the placebo group. The safety outcomes in this study were consistent with those seen with other therapies in large registries of patients with AF, and affirm the need for careful management and selection of haemodynamically stable candidates for cardioversion.

## Abbreviations

ACT, Arrhythmia Conversion Trial; AE, adverse event; AF, Atrial fibrillation; bpm, beats per minute; C.I, confidence interval; CEC, Clinical Events Committee; CHF, chronic heart failure; CYP2D6, Cytochrome P450 2D5; DSMB, Data Safety Monitoring Board; ECG, electrocardiogram; ECV, electrical cardioversion; FDA, Food and Drug Administration; FinCV, Finnish CardioVersion; SAE, serious adverse event; SBP, systolic blood pressure; SD, standard deviation; SPA, Special Protocol Assessment; SR, sinus rhythm.

## Additional file

Additional file 1: List of Ethics Committees and Institutional Review Boards that approved the study. (DOC 234 kb)
