# Supplementary material for: Safety and efficacy of vernakalant for the conversion of atrial fibrillation to sinus rhythm; a phase 3b randomized controlled trial
Source: BMC Cardiovasc Disord. 2016 May 28;16:113. doi: 10.1186/s12872-016-0289-0 (PMC4884402; doi:10.1186/s12872-016-0289-0)
Supplement: Additional file 1: — List of Ethics Committees and Institutional Review Boards that approved the study. (DOC 234 kb) [file 12872_2016_289_MOESM1_ESM.doc]

**Supplementary information.** List of Ethics Committees and Institutional Review Boards that approved the study

| Site # | Principal Investigator | IRB/EC Information |
| --- | --- | --- |
|  |  |  |
| **Canada** | | |
|  | | |
| 0038 | Ian Stiell | The Ottawa Hospital Research Ethics Board  1053 Carling Avenue  Ottawa, ON K1Y 4E9 |
|  |  |  |
| 0054 | Brian Rowe | Health Research Ethics Board Administration Office  308 Campus Tower  8625-112 Street  Edmonton, AB T6G 1K8 |
|  |  |  |
| 0137 | Frank Scheuermeyer | UBC Providence Health Care Research Ethics Board  Providence Health Care Research Institute  Office of Research Services  11^th^ Floor Hornby Site - SPH  c/o 1081 Burrard Street  Vancouver, BC V6Z 1Y6 |
|  |  |  |
| 0165 | Arnold Pinter | St. Michael’s Hospital Research Ethics Board  30 Bond Street  Toronto, ON M5B 1S8 |
|  |  |  |
| 0166 | Jacques Lee | Research Ethics Board of Sunnybrook Health Sciences Centre  Research Ethics Office, Room C8 19  2075 Bayview Avenue  Toronto, ON M4N 3M5 |
|  |  |  |
| 0170 | Thao Huynh | McGill University Health Centre  Biomedical D  Research Ethics Board  Montreal General Hospital  1650 Cedar Avenue  Montreal, QC H3G 1A4 |
|  |  |  |
| 0655 | Steven Clark | University of Calgary  Conjoint Health Research Ethics Board  Office of Medical Bioethics  Room 93, Heritage Medical Research Building  3330 Hospital Drive NW  Calgary, AB T2N 4N1 |
|  |  |  |
| 0666 | Paolo Costi | Comite d’ethique de la Recherche du CHUM  3981 boulevard St-Laurent, Mezz 2  Montreal, QC H2W 1Y5 |
|  |  |  |
| 1222 | Denis-Carl Phaneuf | IRB Services  372 Hollandview Trail, Suite 300  Aurora, ON L4G 0A5 |
|  |  |  |

| Site # | | Principal Investigator | | IRB/EC Information |
| --- | --- | --- | --- | --- |
|  | |  | |  |
| **Canada (cont.)** | | | | |
|  | |  | |  |
| 1229 | | Ian Stiell | | The Ottawa Hospital Research Ethics Board  Ottawa Hospital, Civic Campus  725 Parkdale Avenue, Civic Box 411  Loeb Building  Ottawa, ON K1Y 4E9 |
|  | |  | |  |
| 1485 | | Laurence Sterns | | Vancouver Island Health Authority  Clinical Research Ethics Board  Royal Jubilee Hospital Memorial Pavillion, Kenning Wing 3  1952 Bay Street  Victoria, BC V8R 1J8 |
|  | |  | |  |
| 1833 | | Felix Ayala-Paredes | | Comite d’ethique de la recherché en santé chez l’humain  Centre hospitalier universitaire de Sherbrooke  Hopital Fleurimont  3001, 12 Avenue Nord  Bureau Z5-3014  Sherbrooke, QC J1H 5N4 |
|  | |  | |  |
| 2644 | | Patrick Bergin | | PEI Research Ethics Board  Health Association of PEI  10 Pownal Street  Charlottetown, PEI C1A 3V6 |
|  |  | |  | |
| 2645 | Indraneel Ghosh | | York Central Hospital Research Ethics Board  10 Trench Street  Richmond Hill, ON L4C 4Z3 | |
|  |  | |  | |
| 2646 | Marco Sivilotti | | Queen’s University Health Sciences and Affiliated Teaching Hospitals  Research Ethics Board  Office of Research Services  Room 307 - Fleming Hall - Jemmett Wing  Kingston, ON K7L 3N6 | |
|  |  | |  | |
| 2862 | Paul MacDonald | | Cape Breton District Health Authority  Research Ethics Board  1250 Grand Lake Road  P.O. Box 5300  Sydney, NS B1P 6L2 | |
|  |  | |  | |
| 2863 | Hélène Mayrand | | Comité Scientifique et d’éthique de la recherché du CSSS de Laval  1755 Boul. René Laennec  Laval, QC H7M 3L9 | |
|  |  | |  | |

| Site # | Principal Investigator | IRB/EC Information |
| --- | --- | --- |
|  | | |
| **Chile** | | |
|  |  |  |
| 2581 | Francisco Albornoz | Comité Ético Científico Secretaría Regional Ministerial de Salud VIII region del Bio Bio  Thompson 86  Talcahuano |
|  |  |  |
| 2655 | Pablo Sepulveda | Comité de Ética Científico del Servicio de Salud Metropolitano Oriente  Av. Salvador # 364  Providencia, Santiago |
|  |  |  |
| 2664 | Jorge H. Escobar | Comité Ético Científico del Servicio de Salud Araucania Sur  Andrés Bello # 636  Temuco |
|  |  |  |
| 2716 | Carlos Conejeros | Comité de Ética Científico del Servicio de Salud Metropolitano Sur  Avenida Santa Rosa 3453  San Miguel - Santiago |
|  |  |  |
| 2924 | Guillermo Illanes Brochet | Comité de Ética Científico del Servicio de Salud Metropolitano Oriente  Av. Salvador # 364  Providencia, Santiago |
|  |  |  |
| **Israel** | | |
|  |  |  |
| 1420 | Oscar Kracoff | Institutional Helsinki Committee  Kaplan Medical Center  Rehovot 76100 |
|  |  |  |
| 1421 | Amos Katz | Institutional Helsinki Committee  Barzilai Medical Center  2 Hahistadrout Street  Ashkelon 78278 |
|  |  |  |
| 1427 | David Zeltser | Institutional Helsinki Committee  Tel Aviv Sourasky Medical Center  6 Weitzman Street  Tel-Aviv 64239 |
| 1428 | Tony Hayek | Institutional Helsinki Committee  Rambam Medical Center  8 Haaliya Hashniya Street  Haifa 31096 |
|  |  |  |
| 1430 | Avraham Weiss | Institutional Review Board  Hadassah Medical Organization  Jerusalem 91120 |
|  |  |  |

| **Site #** | **Principal Investigator** | **IRB/EC Information** |
| --- | --- | --- |
|  |  |  |
| **Israel (cont.)** | | |
|  |  |  |
| 1432 | Avraham Shotan | Helsinki Committee  Hillel Yaffe Medical Center  Hadera 38100 |
|  |  |  |
| 1434 | Zvi Vered | Institutional Helsinki Committee  Assaf-Harofeh Medical Center  Zerifin 70300 |
|  |  |  |
| 1435 | Dov Gavish | Institutional Helsinki Committee  Edith Wolfson Medical Center  62 Halochamim Street  Holon 58100 |
|  |  |  |
| 1436 | Mazen Elias | Institutional Helsinki Committee  Emek Medical Center  Afula 18101 |
|  |  |  |
| 2356 | Basil Lewis | Institutional Helsinki Committee  Lady Davis Carmel Medical Center  7 Michal Street  Haifa 34362 |
|  |  |  |
| 2358 | Morris Mosseri | Institutional Helsinki Committee  Meir Medical Center  59 Tchernichovsky Street  Kfar-Saba 44281 |
|  |  |  |
| 2592 | Alon T. Marmor | Institutional Helsinki Committee  Medical Center “Ziv” Safed  Safed 13100 |
|  |  |  |
| 2598 | Nemer Samniah | Institutional Helsinki Committee  Bnai Zion Medical Center  47 Golomb Street  Haifa 31048 |
|  |  |  |
| 2653 | Muhamad Omary | Institutional Helsinki Committee  The Nazareth Hospital E.M.M.S  Nazareth 16100 |
|  |  |  |
| 2669 | Yonathan Hasin | Institutional Helsinki Committee  The Baruch Padeh Medical Center, Poriya  Tiberias 15208 |
|  |  |  |

| **Site #** | **Principal Investigator** | **IRB/EC Information** |
| --- | --- | --- |
|  |  |  |
| **Mexico** | | |
|  | | |
| 2584 | Alberto Z. Banos Velasco | Comité de Ética del Hospital General de Culiacán “Dr. Bernardo J. Gastelum”  Juan Aldama S/N esquina con Nayarit  Col. Rosales. C.P. 80230  Culiacán, Sinaloa |
|  |  |  |
| 2590 | Guillermo A. Llamas Esperón | Comite de Etica Medica e Investigacion del Hospital Cardiologica Aguascalientes  Ecuador 200, Fracc. Las Americas,  Aguascalientes, AGS., 20230 |
|  |  |  |
| 2594 | Gerardo E. Pozas Garza | Comite de Etica del Hospital San Jose Tec  de Monterrey y de la Escuela de Medicina  del Tecnologico de Monterrey  Edificio CITES 3er Piso lado Poniente  Ave. Morones Prieto 3000 Pte., Col. Los Doctores,  Monterrey, N.L., 64710 |
|  |  |  |
| 2596 | Daniel Rodriguez Gonzalez | Comité de Ética del Hospital Civil de Guadalajara “Dr. Juan I. Menchaca”  Salvador Quevedo y Zubieta No. 750  Col. Independencia C.P. 44340  Guadalajara, Jalisco |
|  |  |  |
| 2604 | Jose A. Velasco Barcena | Comité de Etica del Hospital Angeles Puebla  Av. Kepler No. 2143  Col. Reserva Territorial Atlixcayotl, C.P. 72190  Puebla, Puebla |
|  |  |  |
| 2667 | Miguel E. Estrella Garza | Comite de Etica de la Facultad de Medicina de la UANL y Hospital Universitario “Dr. José Eleuterio González”  Av. Francisco I. Madero Pte S/N y Dr. E. Aguirre Pequeño,  Col. Mitras Centro, C.P. 64460  Monterrey, Nuevo León |
|  |  |  |
| 2670 | Manuel R. Barrera Bustillos | Comite de Etica e Investigacion de la Clinica de Merida S.A. de C.V.  Calle 32 No. 242,  Col. Garcia Gineres,  Merida, Yucatan, 97070 |
|  |  |  |
| 2718 | Enrique Lopez Rosas | Comite de Etica del Centro de Especialidades Medicas del Estado de Veracruz “Dr. Rafael Lucio”  Av. Adolfo Ruiz Cortines #2903  Col. Unidad Magisterial  Xalapa, Veracruz  C.P. 91020 |
|  |  |  |

**Appendix 13.1.3 List of IRBs/ECs (page 5 of 13)**

| **Site #** | **Principal Investigator** | **IRB/EC Information** |
| --- | --- | --- |
|  | | |
| **Peru** | | |
|  |  |  |
| 2582 | Ofelia Araoz | Comite de Etica para la Investigacion de la Universidad de San Martin de Porres  Av. Alameda del Corregidor N° 1531 Urb.  Los Sirius, La Molina, Lima 12, Lima |
|  |  |  |
| 2586 | Augusto F. Chois Malaga | Comite de Etica para la Investigacion de la Universidad de San Martin de Porres  Av. Alameda del Corregidor N° 1531 Urb.  Los Sirius, La Molina, Lima 12, Lima |
|  |  |  |
| 2595 | Victor Elías Rodriguez Chávez | Comite de Etica para la Investigacion de la Universidad de San Martin de Porres  Av. Alameda del Corregidor N° 1531 Urb.  Los Sirius, La Molina, Lima 12, Lima |
|  |  |  |
| 2652 | Walter E. Mogrovejo Ramos | Comite de Etica para la Investigacion de la Universidad de San Martin de Porres  Av. Alameda del Corregidor N° 1531 Urb.  Los Sirius, La Molina, Lima 12, Lima |
|  |  |  |
| 2662 | Rodolfo F. Rojas Cañamero | Comite de Etica para la Investigacion de la Universidad de San Martin de Porres  Av. Alameda del Corregidor N° 1531 Urb.  Los Sirius, La Molina, Lima 12, Lima |
|  |  |  |
| 2828 | Luis Camacho Cosavalente | Comite de Etica para la Investigacion de la Universidad de San Martin de Porres  Av. Alameda del Corregidor N° 1531 Urb.  Los Sirius, La Molina, Lima 12, Lima |
|  |  |  |
| 2908 | Aldo E. Rodriguez Escudero | Comité de Bioética de la Red Asistencial Sabogal - ESSALUD  Jirón Colina 1081 Bellavista  Callao 02, Callao |
|  |  |  |
| **South Africa** | | |
|  |  |  |
| 1165 | Johannes S. Roos | South African Medical Association Research Ethics Committee (SAMAREC)  Castle Walk Corporate Park, Block F  Nossob Street  Erasmus Kloof Extension 3  Pretoria, 0153 |
|  |  |  |
| 1848 | Matthys Basson | SAMAREC  Castle Walk Corporate Park, Block F  Nossob Street  Erasmus Kloof Extension 3  Pretoria, 0153 |
|  |  |  |

| **Site #** | **Principal Investigator** | **IRB/EC Information** |
| --- | --- | --- |
|  |  |  |
| **South Africa (cont.)** | | |
|  |  |  |
| 2587 | Iftikhar O. Ebrahim | SAMAREC  Castle Walk Corporate Park, Block F  Nossob Street  Erasmus Kloof Extension 3  Pretoria, 0153 |
|  |  |  |
| 2602 | Nicolaas J. Swanepoel | SAMAREC  Castle Walk Corporate Park, Block F  Nossob Street  Erasmus Kloof Extension 3  Pretoria, 0153 |
|  |  |  |
| 2603 | Louis J. van Zyl | SAMAREC  Castle Walk Corporate Park, Block F  Nossob Street  Erasmus Kloof Extension 3  Pretoria, 0153 |
|  |  |  |
| 2656 | Nicolaas van der Merwe | SAMAREC  Castle Walk Corporate Park, Block F  Nossob Street  Erasmus Kloof Extension 3  Pretoria, 0153 |
|  |  |  |
| 2724 | Colin L. Schamroth | SAMAREC  Castle Walk Corporate Park, Block F  Nossob Street  Erasmus Kloof Extension 3  Pretoria, 0153  Netcare Milpark Hospital Ethics Committee  Milpark Hospital  Guild Road  Parktown West  Johannesburg, 2193 |
|  |  |  |
| 2725 | Leonard Steingo | SAMAREC  Castle Walk Corporate Park, Block F  Nossob Street  Erasmus Kloof Extension 3  Pretoria, 0153 |
|  |  |  |

| **Site #** | **Principal Investigator** | **IRB/EC Information** |
| --- | --- | --- |
|  | | |
| **United States** | | |
|  |  |  |
| 0004 | Gregory Marcus | University of California, San Francisco  Committee on Human Research  3333 California Street, Suite 315  San Francisco, CA 94118 |
|  |  |  |
| 0008 | Linda Eckhardt | Western Institutional Review Board  3535 Seventh Avenue SW  Olympia, WA 98502 |
|  |  |  |
| 0011 | Martin C. Burke | The University of Chicago Institutional Review Board  McGiffert Hall, 2^nd^ floor  5751 S. Woodlawn Avenue  Chicago, IL 60637 |
|  |  |  |
| 0014 | Conor Barrett | Partners Human Research Committee  Human Research Office  116 Huntington Ave, Suite 1002  Boston, MA 02116 |
|  |  |  |
| 0017 | Bruce Koplan | Partners Human Research Office  116 Huntington Avenue, Suite 1002  Boston, MA 02116 |
|  |  |  |
| 0020 | David Benditt | University of Minnesota  Research Subjects’ Protection Program  420 Delaware Street SE, MMC 820  Minneapolis, MN 55455 |
|  |  |  |
| 0053 | Rod Passman | Northwestern University IRB  Rubloff Hall, 7^th^ Floor  750 North Lake Shore Drive  Chicago, IL 60611 |
|  |  |  |
| 0055 | Alexander Green | Loyola University Medical Center  Institutional Review Board for the Protection of Human Subjects  2160 South First Avenue  Maywood, IL 60153 |
|  |  |  |
| 0058 | John M. Wharton | Medical University of South Carolina  Office of Research Integrity  19 Hagood Avenue, Room 601  Charleston, SC 29425 |
|  |  |  |
| 0063 | Ryan Aleong | Western Institutional Review Board  3535 Seventh Ave SW  PO Box 12029  Olympia, WA 98502 |
|  |  |  |

| **Site #** | **Principal Investigator** | **IRB/EC Information** |
| --- | --- | --- |
|  |  |  |
| **United States (cont.)** | | |
|  | | |
| 0071 | Tristram Bahnson | Duke University Health System (DUHS) IRB Office  Hock Plaza - Suite 405  2424 Erwin Road  Durham, NC 27705 |
|  |  |  |
| 0184 | Edward Panacek | UC Davis Institutional Review Board  2315 Stockton Boulevard  Sacramento, CA 95817 |
|  |  |  |
| 0195 | Arnold Greenspon | Thomas Jefferson University  Office of Human Research  1015 Chestnut Street, Suite 1100  Philadelphia, PA19107 |
|  |  |  |
| 0427 | James Daniels | The University of Texas Southwestern Institutional Review Board  5323 Harry Hines Blvd.  Dallas, TX 75390-8843 |
|  |  |  |
| 0463 | Alfred Buxton | Research Protection Office  Rhode Island Hospital Institutional Review Board IRB 2  Aldrich 3  593 Eddy St  Providence, RI 02903 |
|  |  |  |
| 0556 | Soraya Samii | Human Subjects Protection Office/Institutional Review Board  Penn State Milton S. Hershey Medical Center  ASB1140  600 Centerview Drive  Hershey, PA 17033-0850 |
|  |  |  |
| 0559 | John Ip | Ingham Regional Medical Center IRB  401 W Greenlawn Avenue  Lansing, MI 48910 |
|  |  |  |
| 0567 | Scott Pollak | Florida Hospital Institutional Review Board  212 Winter Park Street  Orlando, FL 32804 |
|  |  |  |
| 0667 | Douglas Char | Washington University  Human Research Protection Office  660 S. Euclid Ave., Campus Box 8089  St. Louis, MO 63110 |
|  |  |  |
| 0731 | Peter Levanovich | Northern Michigan Regional Hospital IRB  416 Connable Ave.  Petoskey, MI 49770 |
|  |  |  |

| **Site #** | **Principal Investigator** | **IRB/EC Information** |
| --- | --- | --- |
|  |  |  |
| **United States (cont.)** | | |
|  |  |  |
| 0732 | W. Herbert Haught | Huntsville Hospital Institutional Review Committee  101 Sivley Road  Huntsville, AL 35801 |
|  |  |  |
| 0753 | Steve Rothman | Main Line Hospitals Institutional Review Board  100 Lancaster Avenue  Wynnewood, PA 19096 |
|  |  |  |
| 0878 | Sanders Chae | Western Institutional Review Board  3535 Seventh Ave SW  Olympia, WA 98502-5010 |
|  |  |  |
| 0910 | Karoly Kaszala | McGuire V.A. Medical Center  1201 Broad Rock Blvd  Richmond, VA 23249 |
|  |  |  |
| 1552 | Daniel Lustgarten | Committees on Human Research  Research Protections Office  245 South Park Dr., Suite 900  Colchester, VT 05446 |
|  |  |  |
| 1692 | Jeffery Kluger | Hartford Hospital IRB  80 Seymour Street  Hartford, CT 06102 |
|  |  |  |
| 1790 | Tracy Callister | Schulman Associates IRB  4290 Glendale-Milford Road  Cincinnati, OH 45242 |
|  |  |  |
| 1800 | Robert Winslow | Danbury Hospital Institutional Review Board  24 Hospital Avenue  Danbury, CT 06810 |
|  |  |  |
| 1843 | David Henderson | Schulman Associates IRB  4290 Glendale-Milford Road  Cincinnati, OH 45242 |
|  |  |  |
| 1910 | Brian Olshansky | Western Institutional Review Board  3535 Seventh Ave SW  Olympia, WA 98502 |
|  |  |  |
| 1946 | David R. Burt | Institutional Review Board for Health Sciences Research  One Morton Drive  Suite 400, Box 5  Charlottesville, VA 22903 |
|  |  |  |
| 1970 | Peter Zimetbaum | Committee on Clinical Investigations  Beth Israel Deaconess Medical Center  330 Brookline Ave.  Boston, MA 02215 |
|  |  |  |

| **Site #** | **Principal Investigator** | **IRB/EC Information** |
| --- | --- | --- |
|  |  |  |
| **United States (cont.)** | | |
|  |  |  |
| 2312 | Jalal Ghali | Wayne State University - Human Investigation Committee  101 East Alexandrine  Detroit, MI 48201-2018 |
|  |  |  |
| 2483 | George Horvath | Alta Bates Summit Institutional Review Board  2450 Ashby Avenue  Berkeley, CA 94705 |
|  |  |  |
| 2507 | Dean Kereiakes | The Christ Hospital Institutional Review Board  2139 Auburn Avenue  Cincinnati, OH 45219 |
|  |  |  |
| 2514 | Kisher Vora | Schulman Associates IRB  4290 Glendale-Milford Road  Cincinnati, OH 45242 |
|  |  |  |
| 2529 | Todd Kovach | Baycare Pasco-Pinellas, IRB  207 Jeffords Street  MS 143  Clearwater, FL 33756 |
|  |  |  |
| 2530 | Ameer Kabour | Mercy St. Vincent Medical Center  Adult Institutional Review Board  2213 Cherry St.  Toledo, OH 43608 |
|  |  |  |
| 2539 | Donald Russo | Schulman Associates IRB  4290 Glendale-Milford Road  Cincinnati, OH 45242 |
|  |  |  |
| 2541 | Michael Giudici | Genesis Health System IRB  1227 E. Rusholme St.  Davenport, IA 52803 |
|  |  |  |
| 2542 | Satyaprakash Makam | Community Healthcare System Central Institutional Review Board  901 MacArthur Blvd  Munster, IN 46321 |
|  |  |  |
| 2543 | Otfried Niedermaier | Summa Health System  Institutional Review Board  Research Administration  525 East Market Street  Akron, OH 44304 |
|  |  |  |
| 2557 | Thomas J. Bunch | Intermountain Healthcare Urban Central Region IRB  LDS Hospital  8^th^ Avenue and C Street  Salt Lake City, UT 84143 |
|  |  |  |

| **Site #** | **Principal Investigator** | **IRB/EC Information** |
| --- | --- | --- |
|  |  |  |
| **United States (cont.)** | | |
|  |  |  |
| 2558 | Kevin Browne | Institutional Review Board  Lakeland Regional Medical Center  1324 Lakeland Hills Blvd.  Lakeland, FL 33805 |
|  |  |  |
| 2562 | Matthew Sackett | Schulman Associates IRB  4290 Glendale-Milford Road  Cincinnati, OH 45242 |
|  |  |  |
| 2563 | Hanscy Seide | Liberty IRB  2024 Larchmont Drive  Deland, FL 32724 |
|  |  |  |
| 2564 | Karl Undesser | St. Luke’s Health System Institutional Review Board  190 East Bannock  Boise, ID 83712 |
|  |  |  |
| 2651 | David Law | Institutional Review Board  Saint Francis Medical Center  211 Saint Francis Drive  Cape Girardeau, MO 63703 |
|  |  |  |
| 2715 | Eve Gillespie | Schulman Associates IRB  4290 Glendale-Milford Road  Cincinnati, OH 45242 |
|  |  |  |
| 2717 | Ravi Bhagwat | The Institutional Review Board  St. Margaret Mercy Healthcare Centers  5454 Hohman Avenue  Hammond, IN 46320 |
|  |  |  |
| 2721 | Ofsman Quintana | Schulman Associates IRB  4290 Glendale-Milford Road  Cincinnati, OH 45242 |
|  |  |  |
| 2726 | James DeVille | Baylor Research Institute  Institutional Review Board  3310 Live Oak, Suite 501  Dallas, TX 75204 |
|  |  |  |
| 2727 | Hans Moore | Washington DC VA Medical Center  50 Irving St. NW  Washington, DC 20422 |
|  |  |  |
| 2895 | David Sandler | Hillcrest Medical Center Institutional Review Board  1120 S. Utica Ave.  Tulsa, OK 74104 |
|  |  |  |
